# In Situ Preservation Fraction of Parathyroid Gland in Thyroidectomy: A Cohort Retrospective Study

**DOI:** 10.1155/2018/7493143

**Published:** 2018-03-20

**Authors:** Han Luo, Wanjun Zhao, Hongliu Yang, Anping Su, Bin Wang, Jingqiang Zhu

**Affiliations:** ^1^Thyroid & Breast Surgery, West China Hospital, Sichuan University, Chengdu, China; ^2^Department of Nephrology, West China Hospital, Sichuan University, Chengdu, China; ^3^Biostatistics Center, West China Hospital, Sichuan University, Chengdu, China

## Abstract

**Background and Objectives:**

Parathyroid failure is the most common symptom after thyroidectomy. To prevent it, a gland was preserved in situ or an ischemic one was autotransplanted. This study explored the relationship between in situ preservation of the parathyroid gland and gland failure.

**Methods:**

Consecutive patients who underwent initial total thyroidectomy were enrolled retrospectively in a prospectively maintained database. Patients were divided into groups by parathyroid gland remaining in situ fraction (PGRIF) (PGRIF = number of in situ glands/(total number of identified glands − number of glands in specimen). Patients were graded by tertiles and followed at least one year after surgery.

**Results:**

559 patients were included. PGRIF is significantly inversely associated with transient hypoparathyroidism, protracted hypoparathyroidism, and postoperative hypocalcemia. PGRIF was identified as an independent risk factor for transient hypoparathyroidism, protracted hypoparathyroidism, and postoperative hypocalcemia (OR = 0.177, 0.190, and 0.330, resp.). Autotransplantation of parathyroid gland would not affect the calcium level in the long term.

**Conclusion:**

In situ preservation of parathyroid gland is crucial for parathyroid function. Less preserved is the independent risk factor for postoperative hypoparathyroidism and hypocalcemia, resulting in a worse function of parathyroid gland in the long term.

## 1. Introduction

Hypoparathyroidism is a well-recognized symptom after total thyroidectomy [1–3]. Edafe et al. [4] estimated in a system review that the incidence of transient hypoparathyroidism ranges from 19 to 38 percent and permanent hypoparathyroidism 0 to 3 percent. Multiple factors, including surgery technique, different definitions of hypocalcemia, and calcium or vitamin D supplement, contribute to the wide variation.

Parathyroid function failure is the main cause of postoperative hypoparathyroidism. Preserving all parathyroid glands, vasculature is still challenging, even for high-volume surgeons. Some authors think autotransplantation of discolored parathyroid gland would decrease the incidence of hypoparathyroidism [3, 5–8]; however, it remains controversial [9–12]. In addition, incidental parathyroidectomy frequently happens, in 5.2%–21.6% [13–15], an unnegligible risk factor for postoperative hypoparathyroidism. Therefore, we should consider the effective or functional parathyroid gland fraction when exploring the role of in situ preservation.

In the present designed study, we will explore the association between efficiency of in situ preserved parathyroid gland and parathyroid insufficiency.

## 2. Methods

This retrospective study was conducted based on the database. All databases of patients and surgeries performed by Dr. Zhu were maintained prospectively. All thyroid surgeries were performed by one experienced surgeon (Dr. Zhu), with high volume of over 400 cases annually.

Patients who underwent thyroidectomy in our department between 2013 and 2014 were identified. Inclusion criteria were (1) initial operation and (2) total thyroidectomy (TTx), and exclusion criteria were (1) reoperation, (2) incomplete data, (3) lobectomy and near TTx, (4) any disease that would affect the level of calcium and magnesium (like kidney disease), and (5) patients with preoperative hyper/hypocalcemia.

### 2.1. Surgical Technique

A conventional capsular dissection technique was adopted. Any parathyroid attached on the dorsal side of the thyroid was identified and freed carefully and retained in situ with intact vasculature. If the parathyroid gland was discolored at the end of surgery or was incidentally removed intraoperatively, gland autotransplantation will be performed. Then the parathyroid gland will be chopped into 1 mm^3^ fragments and autografted into the ipsilateral sternocleidomastoid muscle. To avoid incidental parathyroidectomy (IP), we systematically sought to remove the thyroid gland and tissue, in case intrathyroid cases happened. The same pathologist inspected and analyzed all the surgical specimens. After that, the parathyroid gland remaining in situ fraction (PGRIF = number of in situ glands/(total number of identified glands − number of glands in specimen) was graded by tertiles (PGRIF 0–1/3, 1/3–2/3, and 2/3–1): IP = gland  in  specimen + intraoperative  IP. A complete central nodal dissection (CND) was performed, first, on the side of carcinoma.

### 2.2. Perioperative Management

The demographic data about patients and blood (including calcium and intact parathyroid hormone [iPTH]) were collected upon admission. Postoperative calcium and iPTH were measured at 6 AM of the 1st morning. The iPTH level was determined by electrochemiluminescence immunoassay (Roche, USA). Then patients with normocalcemia with no discomfort yet were discharged on the 1st or 2nd postoperative day. The other patients were instituted oral calcium and/or vitamin D supplementation on a case-by-case basis. Supply with calcium and/or vitamin D was decided by the hypocalcemic symptom or biochemical hypocalcemia. On average, each patient received 500 mL total intravenous fluid replacement after surgery until he (she) could drink.

### 2.3. Follow-Up

Patients were followed up rigorously in the outpatient department, from the end of the 1st month after discharge. Patients administrated with calcium and/or vitamin D were followed every month after discharge until 6 months, then 6 months thereafter, or they were followed 1, 3, and 6 month(s) after discharge until 6 months, then 6 months thereafter.

### 2.4. Definition

Transient hypoparathyroidism is defined by a subnormal parathyroid hormone (<1.6 pmol/L), need of calcium replacement after surgery, and absence when followed in the 1st month. Protracted hypoparathyroidism is defined by a persistent subnormal parathyroid hormone (<1.6 pmol/L) and need of calcium replacement with or without calcitriol treatment 4 weeks after surgery. Permanent hypoparathyroidism is defined by a persistent subnormal parathyroid hormone (<1.6 pmol/L) and need of calcium replacement with or without calcitriol treatment 1 year after surgery. Parathyroid insufficiency and hypocalcemia are defined by subnormal parathyroid hormone and serum calcium concentration. The reference is calcium 2.1–2.7 mmol/L and iPTH 1.6–6.9 pmol/L. The Tumor Staging System adopted the standard of the Union for International Cancer Control (UICC) sixth edition.

### 2.5. Statistics

Data analysis was performed by SPSS version 21 (SPSS Inc., Chicago, IL). If normally distributed, continuous variables were presented as the mean ± standard  deviation and compared by *t*-test; if not, variables were presented as median (interquartile range) and compared by *U* test. ANOVA test was used for mean comparison of multigroups. Pearson chi-square test or Fisher's exact test was used to compare frequency (percentage) for categorical variables. Logistic regression was used to determine the risk factor. *P* value < 0.05 indicated significant difference.

### 2.6. Ethics

All experimental protocol in this study involving human participants was approved by the Ethics Committee of West China Hospital, Sichuan University (Chengdu, China). The informed consent forms were obtained from all individual participants in this study. All study participants provided written informed consent to indicate their agreement for the clinical data to be used in clinical research and publication. The methods were carried out in accordance with the Declaration of Helsinki and the guidelines of the Ethical Committee of the Cancer Hospital (Chengdu, China).

## 3. Results

**Figure 1:**
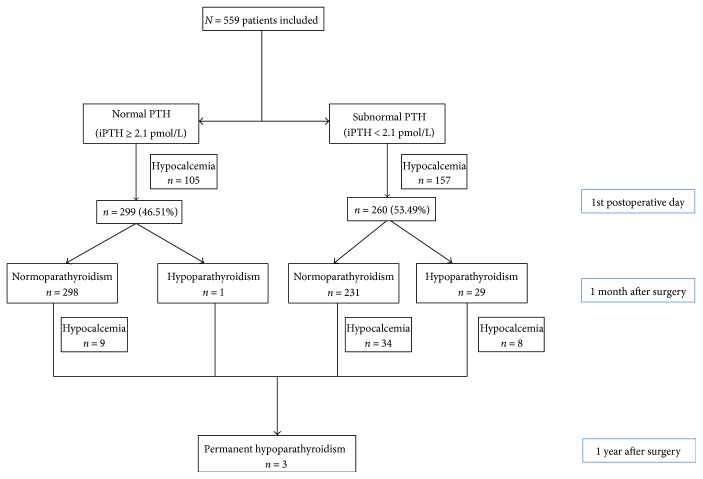
Patient flow chart with function assessment at 1st day, 1st month, and 1 year after total thyroidectomy. iPTH: intact parathyroid hormone.

Based on inclusion and exclusion criteria, 599 patients (428 female) were enrolled in the final analysis, with mean age of 43.25. A total of 238 patients (42.58%) developed transient hypoparathyroidism. 30 patients suffered from protracted hypoparathyroidism (5.37%), and only 3 developed permanent hypoparathyroidism (0.54%) (Figure 1).

Autotransplantation was performed in 357 patients (63.86%, PGRIF 1/2/3: 29/134/194, resp.). An average of 3.55 parathyroid glands were identified (1 gland: 1.43%, 2 glands: 8.94%, 3 glands: 24.51%, 4 glands: 63.33%, and 5 glands: 1.79%). Parathyroid gland was found grossly in 17 specimens (3.04%). Most patients had thyroid carcinoma (539/559).

Significantly more transient hypoparathyroidism patients underwent TT + CND + LND compared with absence of hypoparathyroidism patients (18.46% versus 13.71%, *P* = 0.003). PGRIF showed a significant difference in transient hypoparathyroidism, protracted hypoparathyroidism, and hypocalcemia patients (*P* < 0.001, =0.022, and 0.002, resp.) (Table 1).

**Figure 2:**
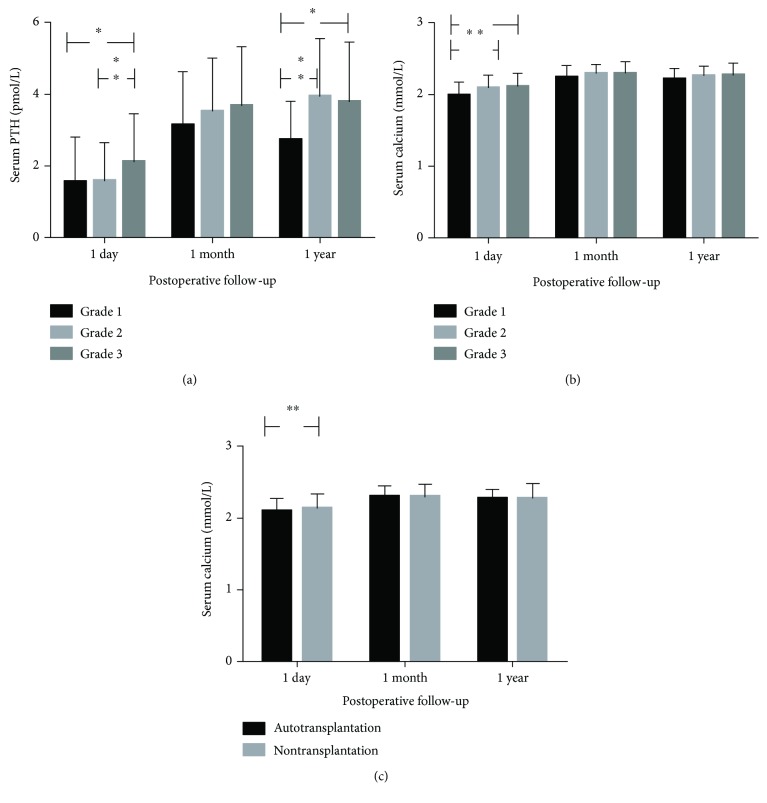
(a) Serum iPTH comparison between grade groups at 1st day, 1st month, and 1 year after total thyroidectomy. Serum iPTH showed significant difference at 1st day and 1 year of follow-up. ^∗^0.001 < *P* < 0.01 and ^∗∗^*P* < 0.001. (b) Serum calcium comparison between grade groups at 1st day, 1st month, and 1 year after total thyroidectomy. Serum calcium showed significant difference at 1st day of follow-up. ^∗^0.001 < *P* < 0.01 and ^∗∗^*P* < 0.001. (c) Serum calcium comparison between autotransplant and nontransplant groups at 1st day, 1st month, and 1 year after total thyroidectomy. Serum calcium showed significant difference at 1st day of follow-up. ^∗^0.001 < *P* < 0.01 and ^∗∗^*P* < 0.001.

There were 31, 136, and 392 patients in PGRIF 1, 2, and 3, respectively. A significant difference was found between PGRIF groups in serum iPTH on the 1st day and 1 year after surgery (*P* < 0.001 and 0.033, resp.). Post hoc analysis showed that the mean of 1st day iPTH after surgery was 1.56 and 1.59 pmol/L in PGRIF 1 and 2, respectively, which was significantly lower than in PGRIF 3 (2.12 pmol/L, *P* = 0.019 and < 0.001, resp.). At 1 year of follow-up, the mean of iPTH in PGRIF 1 (2.73 pmol/L) was still significantly lower than that in PGRIF 2 (3.94 pmol/L) and 3 (3.79 pmol/L), *P* = 0.009 and 0.016, respectively (Figure 2(a)).

In terms of serum calcium level, serum calcium of PGRIF 1 was significantly lower than that of PGRIF 2 and 3 on the 1st day after surgery (*P* = 0.003, PGRIF 1: 1.99 pmol/L, PGRIF 2: 2.09 pmol/L, and PGRIF 3: 2.11 pmol/L) (Figure 2(b)). Specifically, significantly more patients developed hypocalcemia in PGRIF 1 (20/31, 70.97%, *P* = 0.035), comparatively 49.26% (67/136) and 44.13% (173/392) of patients in PGRIF 2 and 3, respectively. This trend was not found at 1 month and 1 year of follow-up.

In addition, mean serum calcium of autotransplantation patients on the 1st day after surgery was significantly lower than that of nontransplantation patients (2.08 versus 2.13 pmol/L, *P* = 0.005). More patients developed hypocalcemia in the autotransplantation group, 180/357, than in the nontransplantation group, 82/202, *P* = 0.028. In contrast, a difference was no longer found at 1 month and 1 year follow-up (Figure 2(c)).

Given only 3 cases with permanent hypoparathyroidism, it is not appropriate for multivariate regression analysis regarding this issue. Therefore, we performed multivariate analysis for transient hypoparathyroidism, protracted hypoparathyroidism, and postoperative hypocalcemia. Sex (male) (OR = 0.577, *P* = 0.016) and PGRIF (OR = 0.177, *P* < 0.001) were identified as independent risk factors for transient hypoparathyroidism, while only PGRIF (OR = 0.190, *P* = 0.030) was identified as a risk factor for protracted hypoparathyroidism. Regarding postoperative hypocalcemia, sex (male) (OR = 0.472, *P* = 0.001) and PGRIF (OR = 0.330, *P* = 0.006) were identified as risk factors (Table 2).

## 4. Discussion

Since thyroidectomy became a popular surgical option for most thyroid entities in the 1980s, postoperative complications are besetting surgeons and physicians. Postoperative hypocalcemia and hypoparathyroidism are the most common complications after total thyroidectomy. Patients' age, preoperative serum calcium, gender, and extent of surgery are associated with postoperative hypocalcemia [10, 16–19]. Our present study largely confirmed the findings in previous studies, yet the preserving gland in situ fraction seemed more overwhelming than did other variables, which confirmed the importance of preservation in situ as in previous researches [9, 12, 20].

However, we adopted the concept of relative fraction of parathyroid gland preserved in situ in our study, not absolute number. In agreement with a previous study, 5% of the individuals had fifth parathyroid gland after cadaveric dissection of 942 patients [21]. In the present study, 1.79% of patients in our study had 5 glands. Therefore, we thought it more reasonable to consider the baseline when considering the efficiency of in situ preservation.

In our study, less in situ preserved parathyroid gland led to lower serum iPTH (*P* < 0.001) and calcium (*P* = 0.003) on the 1st day after surgery significantly. Song et al. [7] concluded that preservation of all parathyroid glands decreases transient hypoparathyroidism compared with that when three or fewer glands are preserved. Less preservation means more autotransplantation. Therefore, we also found that autotransplantation of parathyroid gland is predisposed to immediate postoperative hypocalcemia (*P* = 0.028), consistent with Hallgrimsson et al.'s [10] and Kihara et al.'s [20] previous research.

No significant difference appeared in both calcium and iPTH level 1 month after surgery. El-Sharaky et al. [1] considered that parathyroid function would recover gradually from the 2nd week to normal level on the 4th week after surgery after blood test and electron microscopy analysis. However, patients in PGRIF 1 had significantly lower iPTH than PGRIF 2 and 3 patients had (2.73 pmol/L versus 3.94 pmol/L and 3.79 pmol/L, resp.) in the 1-year follow-up. Similarly, Kihara et al. [20] found that autotransplantation produces inadequate recovery of long-term function after a 5-year follow-up. It may indicate that the surgeon should preserve all parathyroid glands, regardless of their appearance. The parathyroid gland remaining in situ fraction (PGRIF) in our study had an impact on function recovery of parathyroid gland in the long term.

As we know, extent of surgery has a well-established relation with hypocalcemia after thyroidectomy [11, 19, 22, 23]. In our study, more transient hypoparathyroidism patients had TT + CND and LND, though it was not identified as an independent risk factor. It is well known that CND is a widely accepted risk factor for postoperative hypocalcemia and hypoparathyroidism [24]. In situ preservation outweighs surgery extent alone. Therefore, tremendous effort should be made to preserve gland in situ.

Autotransplantation of parathyroid glands was performed in 357 patients (63.86%), which is higher than previous results at 15%–50% [1, 22, 25]. Our place of study was a tertiary institute and most referred patients had thyroid carcinoma; only a small part had large goiters. So, CND is performed in most included patients. As we know, lymph node dissection is a pivotal risk factor for intraoperative incidental removal of glands [15]. Therefore, when incidental resection happened intraoperatively, we performed autograft, which led to a relatively high ratio of autotransplantation.

Our research was limited by its retrospective nature, though the database was maintained prospectively. First, patient collection bias was unavoidable because over 95% of patients have malignancy, which resulted from the referring system. Second, as mentioned before, a high proportion of malignancy in the present study led to a large quantity of CND, so autotransplantation of parathyroid glands was performed in 357 patients. Therefore, we plan to conduct a multicenter retrospective study of recruiting more benign thyroid goiter patients to confirm this result. In addition, there lacked complete data about preoperative 25-hydroxy vitamin D (25OH-VD) level, so we did not take it into the final analysis. Yet, current meta-analysis evidence showed that lower preoperative 25OH-VD would not increase risk of transient hypocalcemia [4, 26].

In conclusion, in situ preservation of parathyroid glands seems crucial for gland function. Though autotransplantation would not affect serum calcium in the long term, more gland preserved in situ was beneficial for gland function in the follow-up. Female patients with less parathyroid gland preserved in situ were vulnerable in transient hypoparathyroidism and postoperative hypocalcemia, and PGRIF was identified as a sole risk factor for transient hypoparathyroidism, protracted hypoparathyroidism, and postoperative hypocalcemia. At least preserving 3 parathyroid glands is a better way to avoid postoperative complications. Further prospective research with long-term follow-up is needed to confirm this result.

## Figures and Tables

**Table 1:** Influence of clinical variables, extent of surgery, and in situ preservation of parathyroid gland on postoperative hypocalcemia and transient and protracted hypoparathyroidism.

	Transient hypoparathyroidism			Protracted hypoparathyroidism			Postoperative hypocalcemia		
	Yes	No	*P*	Yes	No	*P*	Yes	No	*P*
Age	43.48 ± 12.88	43.04 ± 12.84	0.688	40.67 ± 13.53	43.39 ± 12.81	0.259	42.99 ± 12.72	43.47 ± 12.98	0.662
Sex (male/female)	52/208	79/220	0.089	5/25	126/403	0.507	45/217	86/211	0.001
Hypertension	26/234	31/268	>0.999	2/28	55/474	0.758	27/235	30/267	1.000
Diabetes	6/254	6/293	0.774	0/30	12/517	>0.999	9/253	3/294	0.076
HD	71/189	92/207	0.401	8/22	155/374	0.839	78/184	85/212	0.780
Preoperative PTH	5.61 ± 2.94	5.80 ± 2.19	0.386	4.95 ± 0.10	5.75 ± 2.51	0.104	5.55 ± 2.27	5.85 ± 2.79	0.165
Preoperative calcium	2.35 ± 0.40	2.33 ± 0.26	0.555	2.34 ± 0.14	2.34 ± 0.34	0.928	2.32 ± 0.27	2.35 ± 0.38	0.265
Extent of surgery			0.030			0.173			0.981
TTx	3	12		1	14		8	7	
TTx + CND	209	246		21	434		212	243	
TTx + CND + LND	48	41		8	81		42	47	
PGRIF			<0.001			0.022			0.002
0–1/3	21	10		6	25		22	9	
1/3–2/3	76	60		8	128		67	69	
2/3–1	163	229		20	372		173	219	

HD: Hashimoto disease; PTH: parathyroid hormone; TTx: total thyroidectomy; CND: central nodal dissection; LND: lateral nodal dissection; PGRIF: parathyroid gland remaining in situ fraction.

**Table 2:** Independent risk factor identification of postoperative hypocalcemia and transient and protracted hypoparathyroidism.

	Odds ratio	95% CI	*P* value
Transient hypoparathyroidism			
Sex (m/f)	0.577	0.369, 0.901	0.016
TTx + CND + LND	4.918	0.962, 25.150	0.056
PGRIF	0.177	0.079, 0.398	<0.001
Postoperative hypocalcemia			
Sex	0.472	0.308, 0.722	0.001
PGRIF	0.330	0.149, 0.728	0.006
Preoperative PTH	0.938	0.874, 1.007	0.078
Protracted hypoparathyroidism			
PGRIF	0.190	0.042, 0.854	0.030
Preoperative PTH	0.838	0.673, 1.045	0.116

TTx: total thyroidectomy; CND: central nodal dissection; LND: lateral nodal dissection; PGRIF: parathyroid gland remaining in situ fraction; PTH: parathyroid hormone.
